# Development of Wearable Sheet-Type Shear Force Sensor and Measurement System that is Insusceptible to Temperature and Pressure

**DOI:** 10.3390/s17081752

**Published:** 2017-07-31

**Authors:** Shigeru Toyama, Yasuhiro Tanaka, Satoshi Shirogane, Takashi Nakamura, Tokio Umino, Ryo Uehara, Takuma Okamoto, Hiroshi Igarashi

**Affiliations:** 1 Research Institute, National Rehabilitation Center for Persons with Disabilities, 4-1 Namiki, Tokorozawa, Saitama 359-8555, Japan; tanaka-yasuhiro@shiewatech.jp (Y.T.); shirogane-satoshi@rehab.go.jp (S.S.); nakamura-takashi@rehab.go.jp (T.N.); 2 Department of Electronic Engineering, School of Engineering, Tokyo Denki University, Tokyo 120-8551, Japan; t.umino@crl.epi.dendai.ac.jp (T.U.); r.uehara@crl.epi.dendai.ac.jp (R.U.); t.okamoto@crl.epi.dendai.ac.jp (T.O.); h.igarashi@mail.dendai.ac.jp (H.I.)

**Keywords:** shear force sensor, liquid electrolyte, flexible electrode film, mobile sensor system, compensation calculation, wheelchair, prosthetics

## Abstract

A sheet-type shear force sensor and a measurement system for the sensor were developed. The sensor has an original structure where a liquid electrolyte is filled in a space composed of two electrode-patterned polymer films and an elastic rubber ring. When a shear force is applied on the surface of the sensor, the two electrode-patterned films mutually move so that the distance between the internal electrodes of the sensor changes, resulting in current increase or decrease between the electrodes. Therefore, the shear force can be calculated by monitoring the current between the electrodes. Moreover, it is possible to measure two-dimensional shear force given that the sensor has multiple electrodes. The diameter and thickness of the sensor head were 10 mm and 0.7 mm, respectively. Additionally, we also developed a measurement system that drives the sensor, corrects the baseline of the raw sensor output, displays data, and stores data as a computer file. Though the raw sensor output was considerably affected by the surrounding temperature, the influence of temperature was drastically decreased by introducing a simple arithmetical calculation. Moreover, the influence of pressure simultaneously decreased after the same calculation process. A demonstrative measurement using the sensor revealed the practical usefulness for on-site monitoring.

## 1. Introduction

Thin wearable shear force sensors have been desired in the field of rehabilitation engineering. One example is a sensor used to measure the shear force between the prosthetic limb and stump of lower limb amputee patients. The compatibility of prosthetic limbs with stumps currently depends on the intuition and experience of the prosthetist and the feeling of the user’s fit. Therefore, tools to obtain objective information to decide on compatibility are desired. There have been trials to incorporate force sensors in prosthetics. For example, Sanders et al. [1] incorporated a force sensor in a prosthetic limb; however, the sensor was bulky and was installed with a hole in the prosthetic foot. Although this method proved interesting as a piece of research, it cannot be applied to prosthetic limbs used by patients.

Another example is a measurement of the shear force between the buttocks and the seat of a wheelchair, as not only pressure, but also shear force are considered to be causes of pressure ulcers [2,3,4]. One simple method of measuring the shear force on a wheelchair is to use force plates [5,6]. Since force plates have a relatively large area, they can measure the total shear force. Attempts have also been made to place small sensors on the seating surface to investigate so-called local shear force in detail. Bennett et al. [7] placed several spot sensors—including a shear force sensor—on the seat of a wheelchair, as did Goossens et al. [8], who also used an array of sensors on the wheelchair seat. Although such research is pioneering, studies that attach a sensor to the body side has still to be pursued. If a thin and flexible sensor is provided, measurements could be performed where a sensor is stuck on clothing corresponding to the part of attention, such as the sacrum or ischial tuberosity of the human body.

In the literature, some shear force sensors measure the shear force at the interface between the sensor and the fluid in contact with it [9,10,11], though they do not cover our requirements. The sensor required by our study should measure the shear force between two solids by inserting it between the solids. According to the literature survey, various types of shear force sensors have been developed, many of which have already been commercialized. One type uses a photoelectronic device to detect the deviation between the upper and lower sides [12]. Another uses piezoresistive detectors [13,14,15,16,17,18,19]. We can also find capacitive detectors [20,21,22]; however, the size of most conventional sensors is either not compact enough, or their thickness too great.

**Figure 1:**
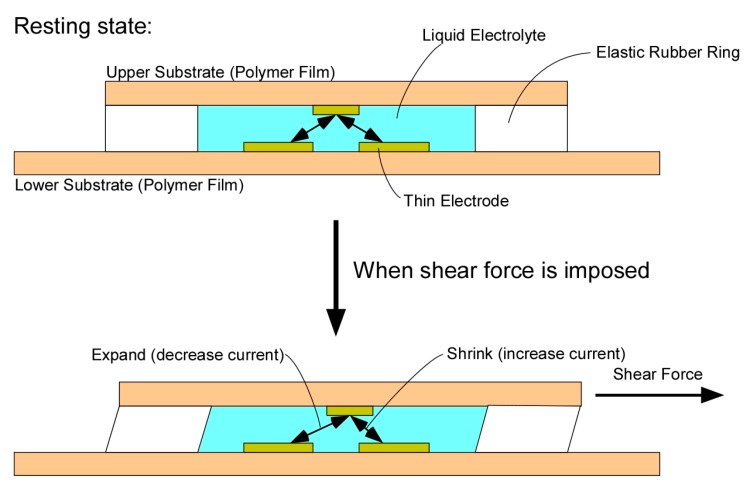
Schematic representation of the operating principle of the shear force sensor. The pictures show the side view of the sensor in the resting state and the shear force-imposed state.

**Figure 2:**
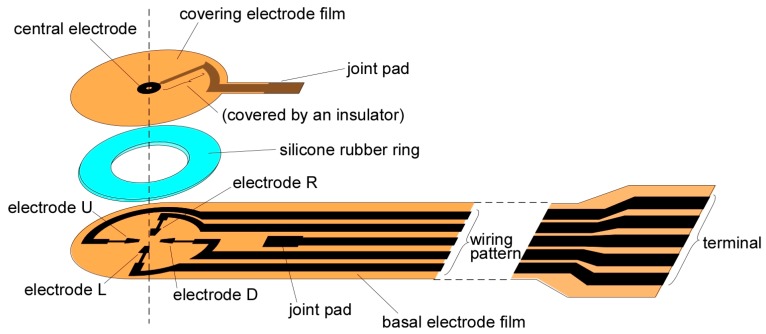
Schematic representation of the structure of the shear force sensor.

To date, we have been developing a shear force sensor composed of only soft materials such as flexible film electrode (capable of bending, but not stretchable), elastic rubber, and liquid electrolyte [23]. One of the advantages of using liquid electrolyte is that the contact resistance at the surface of the electrode is stable because the electrolyte does not peel off from the electrode even after repeated deformation. Recently, several types of tactile sensors using a liquid conductor have been developed. Some use ionic liquid [24,25], and others use metal liquid [26,27]. In our case, we used liquid electrolyte, as the conductivity is easily controllable by simply changing the concentration of the ionic species. In this case, if a voltage is applied while the electrode and the liquid are in contact with each other, an electrochemical reaction occurs on the surface of the electrode, and the electrode may be oxidized and deteriorate, or bubbles may be generated. However, this problem has been avoided by devising a method of applying liquid material and voltage [23], but the sensor had a somewhat complicated structure and its thickness was 2 mm, which was not thin enough. Therefore, we simplified the structure of the sensor shown in Figure 1 (the conceptual structure with a slight difference was presented in the domestic symposium [28]). Here, the sensor had essentially two electrode films: the covering and basal electrode films (Figure 2). The covering film had an electrode (central electrode) at the center, and the basal film had four electrodes that were arranged point-symmetrically. There was an elastic silicone rubber ring sandwiched between the two electrode films, and the space formed by the two films and the rubber ring was filled with a liquid electrolyte. When a shear load was imposed on the surface of the sensor, the rubber ring deformed, resulting in the distance between the upper electrode and the lower electrode changing. As a result, the current flowing between the upper electrode and the lower electrode increases or decreases. In this case, the thickness was improved to about 0.7 mm.

At this stage, the major problem remaining was that the raw sensor output was affected by temperature. Nevertheless, the dependency on temperature was remarkably improved by introducing a simple arithmetical calculation. Hence, we no longer needed to add a temperature sensor (such as a thermistor) to compensate for the temperature dependence of the shear force sensor. In this paper, we detail the sensor structure, measurement system, and compensation method.

## 2. Materials and Methods 

### 2.1. Fabrication of Sensor Device

The fabrication process of the sensor (Figure 2) is explained as follows. The patterning procedure of the electrode film was followed by our previously published paper [29], and is described briefly as follows. The basal polymer film of the electrode was polyimide (Kapton^®^, Du Pont-Toray Co., Ltd. (Tokyo, Japan)), thickness: 50 μm. A reversal pattern of the electrodes was printed on the film with a laser printer (LP-S5300, Seiko Epson Co. (Suwa, Japan)). Then, multi-metal layers were deposited on the film with an electron beam evaporator (EBX-8C, ULVAC Inc. (Chigasaki, Japan)). The evaporated metal layers were Ni, Ag, and Au, and their thicknesses were 200, 200, and 100 nm, respectively. After evaporation, the film was dipped into acetone and sonicated to remove toner and metal layers (i.e., a kind of lift-off process). In addition, the film was manually cut in the form with a cutter. The diameter of the sensor head was 10 mm. Following this, the wiring pattern of the covering film (from the central electrode to the edge) was insulated with an adhesive agent.

The silicone rubber ring was obtained from a silicone rubber film (SR-50, Tigers Polymer Co. (Toyonaka, Japan), thickness: 0.5 mm) using a cutting plotter (CE6000-40, Graphtec Co. (Yokohama, Japan)). The outer diameter of the ring was 9.0 mm, and the inner diameter was 5.6 mm. The outer diameter was slightly smaller than that of the electrode films to prevent any leakage of the adhesive agent to the surroundings in the subsequent process.

The liquid electrolyte was 30 mM LiCl in ethylene glycol. The component was similar to that of our previously reported sensor [23], but the concentration of LiCl was different. The main reason that this liquid was used was to prevent electrode damage due to the electrochemical reaction. The electrolyte was heated at 110 °C under reduced pressure for about 1 h to remove air bubbles just before use.

The rubber ring and the electrode-patterned film were adhered using a mixture of adhesive agents as there was no best single adhesive agent for both silicone rubber and polyimide. We used a silicone-affinitive adhesive agent (Bathcoke N, Cemedine Co. (Tokyo, Japan)) and a polyimide-affinitive adhesive agent (AX-016, Cemedine Co. (Tokyo, Japan)) at a mixture ratio of almost 1:1, and they were mixed just before use.

The terminals and the wiring patterns from the electrodes to the terminals were also fabricated on the basal film. The wiring pattern from the edge of the covering film was finally firmly connected to that of the basal film by soldering. The soldering position was designed slightly apart from the sensor head so that the relative movement of the covering and the basal films was not constrained.

### 2.2. Measurement System

**Figure 3:**
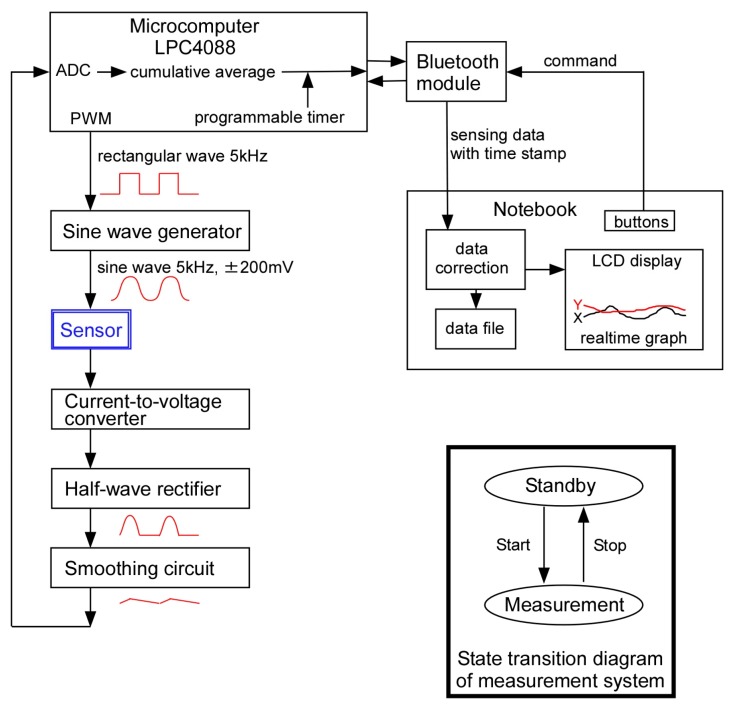
Block diagram of the measurement system. ADC: analog to digital converter; PWM: pulse width modulator.

Figure 3 shows the block diagram of the measurement system. The major components of the system were a mobile measurement circuit and a notebook computer. The measurement circuit was composed of a microcomputer-equipped small board system (LPC4088 QuickStart Board, Embedded Artists AB (Malmo, Sweden)) with a Bluetooth module (RN42XVP-I/RM, Microchip Technology Inc., Chandler, AZ, USA) and analog components. We used this as a base given its many useful functions, such as being an analog to digital converter (ADC), a floating-point calculator, an internal timer, a pulse width modulator (PWM), and a flash memory.

When driving the sensor, an alternating voltage (5 kHz, –200 to +200 mV) was applied between the electrodes to minimize irreversible damage on the thin film electrodes due to the electrochemical reaction. To obtain a sinusoidal wave, a rectangular wave of 5 kHz was generated from the PWM, and the wave was modified to a sine wave with an analog circuit. The output current of the sensor was then converted to a voltage signal, rectified, smoothed, and converted to a digital signal with the ADC. Finally, the digital signal was accumulated and averaged to gain the signal-to-noise ratio and submitted to the notebook computer via Bluetooth telecommunication. For convenience, a USB mobile power module was used as the power source of the measurement circuit.

### 2.3. Computer Program

A specialized computer program with a graphical interface was developed and installed on a notebook computer (OS: Windows 10) and was written by C# language of Visual Studio 2015 (Microsoft Co. (Redmond, WA, USA)). The program received the sensor data from the measurement circuit via Bluetooth wireless communication, applied mathematical calculations to the raw sensor data to stabilize the baseline, displayed data as a graphical plot, and stored the data as a file. 

Subsequently, after receiving the data from the mobile measurement system to the computer, the data was accumulated and averaged again in the computer. At this point, the data is referred to as “raw data”. Since the sensor had four electrodes that represented orientations, raw data from four electrodes were obtained simultaneously. As part of the data compensation process, the raw data were normalized as per the following simple arithmetical calculations.

NORM_L_ = RAW_L_/(RAW_L_ + RAW_R_ + RAW_U_ + RAW_D_), (1)

NORM_R_ = RAW_R_/(RAW_L_ + RAW_R_ + RAW_U_ + RAW_D_), (2)

NORM_U_ = RAW_U_/(RAW_L_ + RAW_R_ + RAW_U_ + RAW_D_), (3)

NORM_D_ = RAW_D_/(RAW_L_ + RAW_R_ + RAW_U_ + RAW_D_), (4)

where RAW_X_ and NORM_X_ are the raw data and the normalized data corresponding to the electrodes, respectively.

In the final process, the normalized data were processed by the following equations and then displayed and stored as a computer file.

Force_x_ = k_x1_ (NORM_R_ − NORM_L_) + k_x2_, (5)

Force_y_ = k_y1_ (NORM_U_ − NORM_D_) + k_y2_, (6)

where k_x1_, k_x2_, k_y1_, k_y2_ are the characteristic values calculated from the calibration curves obtained from the actual response of each sensor.

### 2.4. Testing Apparatus

The temperature dependency of the sensor was evaluated by a thermo-hygrostat (Isuzu Seisakusho Co., Ltd., (Sanjo, Japan) TPAV-48-20). While the temperature was changed by the sequence controller of the thermo-hygrostat, humidity was constantly kept at 40% throughout the evaluation.

**Figure 4:**
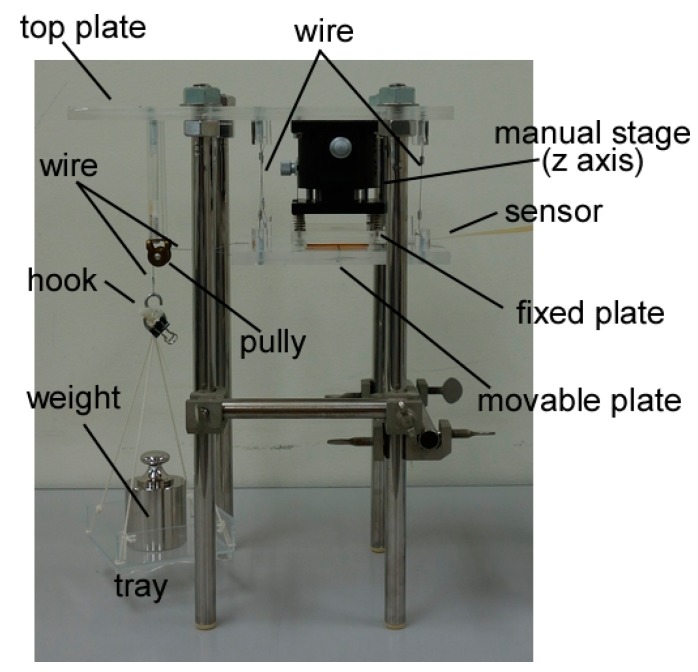
Testing apparatus to obtain sensor output vs. shear load.

A shear force application test was performed using a self-built testing apparatus (Figure 4) with a fixed plate and a movable plate. These plates were parallel and horizontally disposed. The distance between them was adjustable so that the sensor was pinched between them. A wire was connected to the movable plate, and the other end of the wire was passed through a pulley and suspended a tray. Shear force was applied by loading a weight on the tray.

A pressure application test was performed by simply loading a weight on the sensor while the gravity center of the weight was carefully placed at the center of the sensor head. The weight was not in direct contact with the sensor, but a glass plate of 1 mm thickness was inserted.

### 2.5. Test Subjects

We did two kinds of experiment using test subjects. All subjects gave their informed consent for inclusion before they participated in the study. The study was conducted in accordance with the Declaration of Helsinki, and the protocol was approved by the Ethics Committee of the National Rehabilitation for Persons with Disabilities (Identification code: 29-41 and 28-191).

## 3. Results

**Figure 5:**
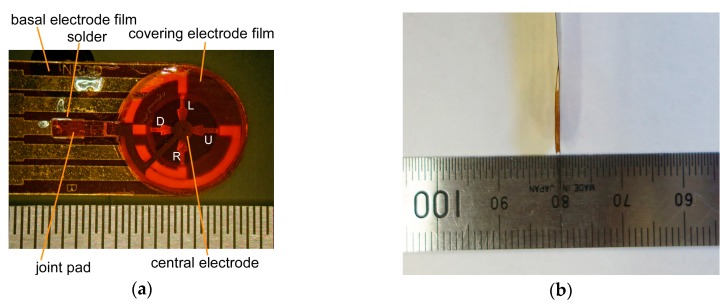

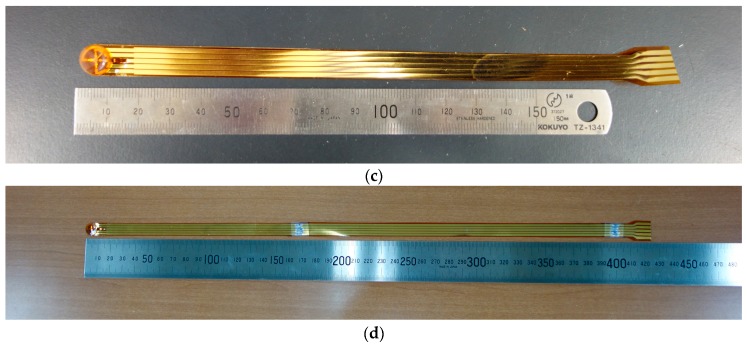
Photographs of the sensor: (**a**) Frontal view of the head; (**b**) Side view of the head; (**c**) Standard sensor outlook; and (**d**) Elongated sensor outlook.

Photographs of the sensor are shown in Figure 5. The diameter of the sensor head (diameter of the covering electrode film) was about 10 mm, as previously stated. The actual thickness of the sensor was about 0.7 mm measured with a caliper, though the total thickness of materials was 0.6 mm. The left side silver bright parts in Figure 5a were solders attributed to the unintended contact of soldering iron during the bonding work of pads of basal and covering films. The soldering tightly bonded the covering and the basal electrode films, and problems such as cracks were not found. It was hard to peel the two films from each other. The length of the wiring pattern was about 17 cm, except for the terminal part due to the spatial limitation of the chamber size of our evaporator (see Figure 5c). However, it was possible to elongate it by at least 40 cm by soldering another wiring-patterned film (see Figure 5d). The thickness of the junctions did not exceed 0.6 mm, including the thickness of the protecting tape.

One notable issue was that a problem arose if there was poor fabrication of the sensor. It was conceivable that the sensor collapsed through leakage of the internal electrolyte; however, this could be easily recognized from the abnormal fluctuation of the baseline of the sensor signal. Additionally, the leakage of liquid was immediately noticed after the end of the measurement. It was easily noticed by the naked eye, and was also confirmed by the weight change of the sensor before and after use. In practice, however, such problems rarely happen now that the fabrication technology has been established.

**Figure 6:**
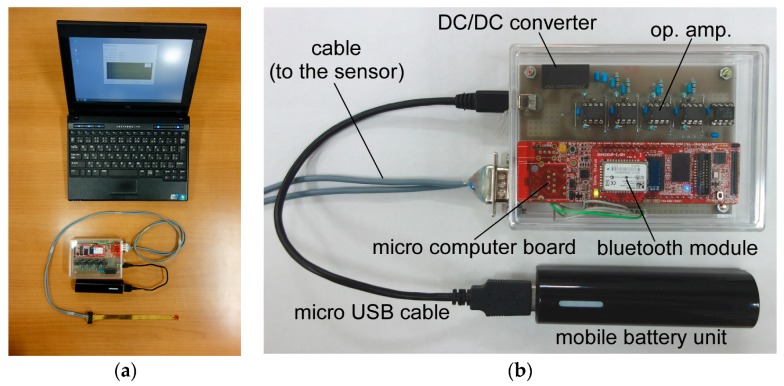
Measurement system for the shear force sensor: (**a**) Whole system; and (**b**) Expanded photograph of the measurement circuit.

Figure 6 is a photograph of the measurement system. The circuit board was placed in a see-through box. We used a plastic box so that radio waves could pass through it. The size of the box was 110 mm × 80 mm × 33 mm. The weight of the circuit including the box was 123 g, and the total weight of the mobile system including the cables, battery, and the sensor was 231 g. The circuit consumed about 230 mA (at 5.0 V) in the maximum scenario. Therefore, the system could continuously work for more than eight hours when the capacity of the mobile battery was 9.6 Wh.

**Figure 7:**
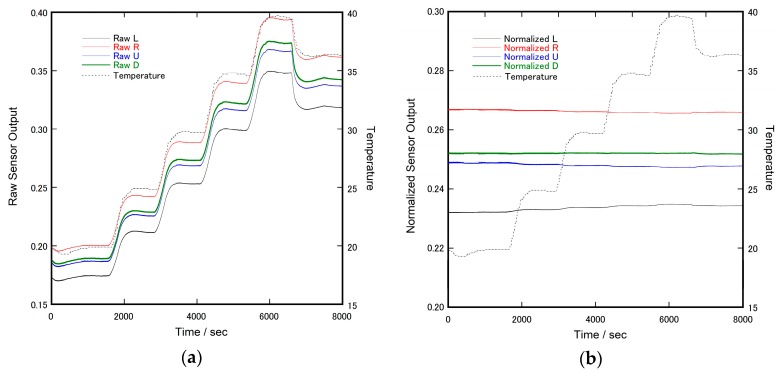
Time courses of the sensor output and the chamber temperature of the thermo-hygrostat: (**a**) Raw sensor output. The vertical scale of raw data is equivalent to the signal level of ADC (full range is 0.0–3.3 V); and (**b**) Normalized sensor output (no unit).

Figure 7 shows the time courses of the raw sensor output and that of the normalized output under temperature variation control. The raw sensor output was considerably influenced by temperature, while the normalized output was almost independent of it.

**Figure 8:**
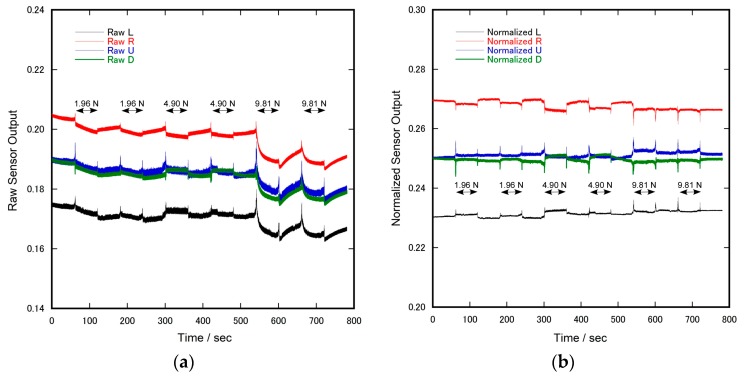
Time courses of the electrode output during the intermittent application of pressure (normal force): (**a**) Raw sensor output. The vertical scale of raw data is equivalent to the signal level of ADC (full range is 0.0–3.3 V); and (**b**) Normalized sensor output (no unit).

Furthermore, the same normalization procedure also stabilized sensor output upon the application of pressure. Figure 8 shows the time courses of the raw sensor output and that of the normalized output during the intermittent application of pressure (normal force). In this case, the raw sensor output was influenced by pressure, while that of the normalized output was minor.

**Figure 9:**
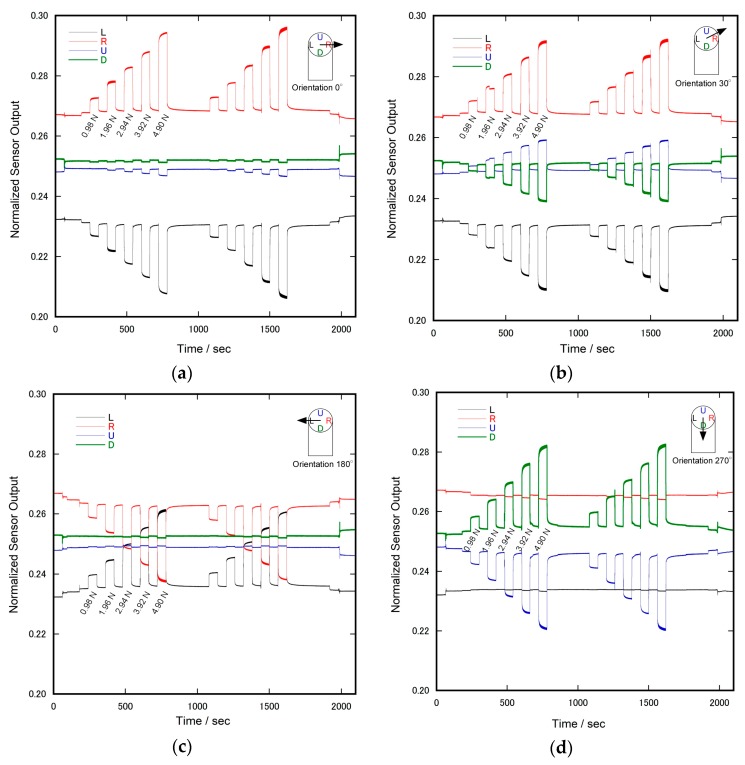

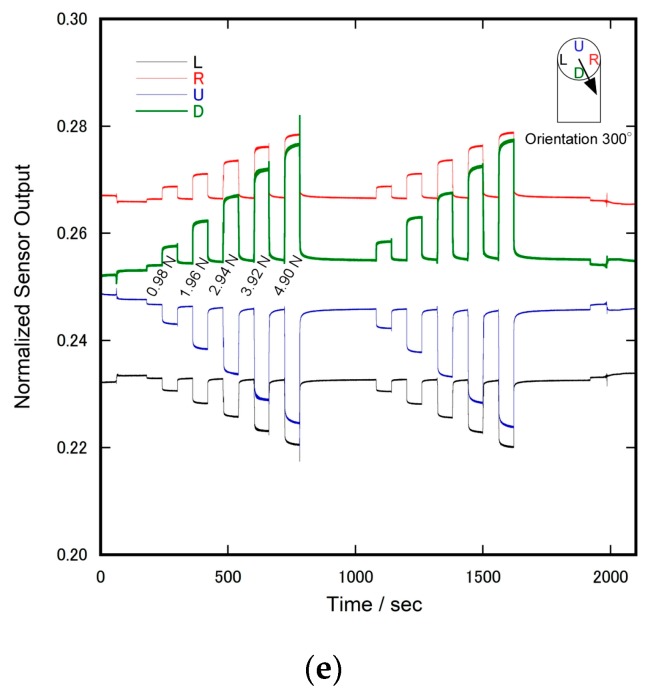
Time course of normalized sensor output during the application of shear force: The orientations of applied shear force were (**a**) 0; (**b**) 30; (**c**) 180; (**d**) 270; and (**e**) 300 degrees, respectively. The magnitude of the shear force was intermittently changed, and the same pattern of the application was repeated twice in each testing.

Figure 9 shows the time courses of the normalized sensor output during the application of shear force intermittently changing the magnitude. In each measurement, the following events were performed. After just 1 min from the beginning of the measurement, the sensor was pinched by the upper and lower plates of the testing apparatus. A slight decrease or increase of the sensor output (seen in each graph) was attributed to this event. After two minutes, a vacant tray was suspended from the edge of the wire of the shear force testing apparatus. One minute later, a weight was placed on the tray. Another minute later, the weight was taken off the tray, and repeated afterward. After 5 min from the removal of the last weight, the vacant tray was removed from the testing apparatus, and after one more minute, the sensor was released from pinching.

As seen in Figure 9, the sensor output exhibited a large and rapid response upon the application of shear force and a relatively small but long-term response. After releasing the shear force, a rapid recovery and a small but gradual recovery was also observed. Since even the long-term change almost ceased after a minute, it was possible to estimate the magnitude of the sensor response to the magnitude of applied shear force.

**Figure 10:**
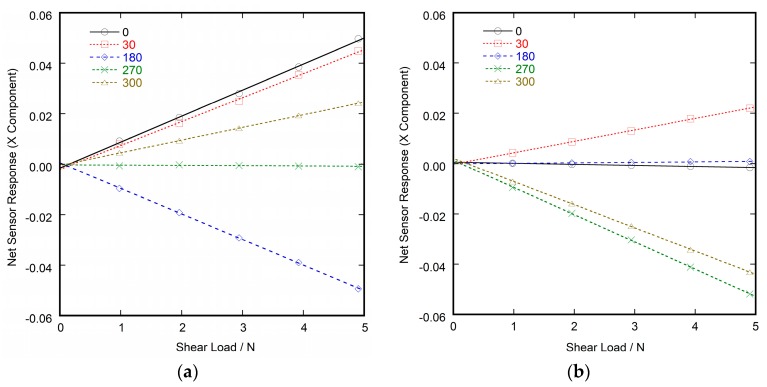
Relation between the component expression of the sensor output and the magnitude of the applied shear force. The data shown in this figure were calculated from the data in Figure 9. Each line corresponds to the shear force with different direction angles. The lines are the results of least squares fitting. The number of each legend indicates the direction angle. (**a**) Shear force component of x-direction; and (**b**) Shear force component of y-direction.

Figure 10 shows the calibration curves of the net sensor response (defined as the difference between the response and baseline) calculated from the data shown in Figure 9. In this figure, the net sensor response (NSR) was represented as x and y components (NSR_X_ and NSR_Y_), which are calculated by the following equations;

NSR_X_ = (NORM_Ra_ − NORM_Rb_) − (NORM_La_ − NORM_Lb_), (7)

NSR_Y_ = (NORM_Ua_ − NORM_Ub_) − (NORM_Da_ − NORM_Db_), (8)

where NORM_Ra_ is the value of the normalized sensor output corresponding to the R electrode (NORM_R_) just before the release of loading, and NORM_Rb_ is that just before the application of loading, and also NORM_La_, NORM_Lb_, NORM_Ua_, NORM_Ub_, NORM_Da_, and NORM_Db_. The relationship between the sensor output and the shear load was almost proportional (as shown in Figure 10). This result suggests that the principle of operation of the sensor (Figure 1) was appropriate. Consequently, shear force can be estimated from the output of the sensor by a linear equation. Thus, we used Equations (5) and (6) to find the shear force from the sensor output obtained in the application experiments.

**Figure 11:**
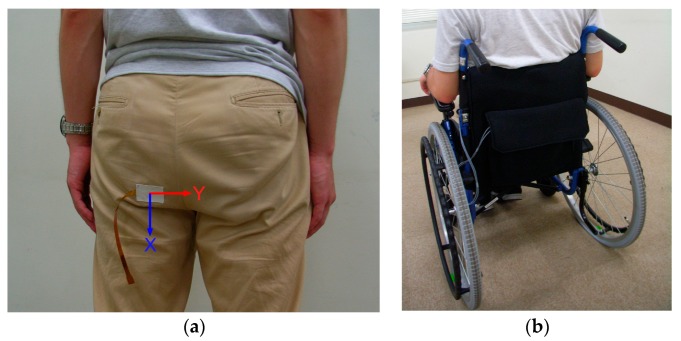
Landscape of a demonstrative measurement using a wheelchair: (**a**) Shear force sensor was attached to the trouser cloth; and (**b**) Experimental subject sat in a wheelchair. The mobile measurement system was housed in the rear pocket.

**Figure 12:**
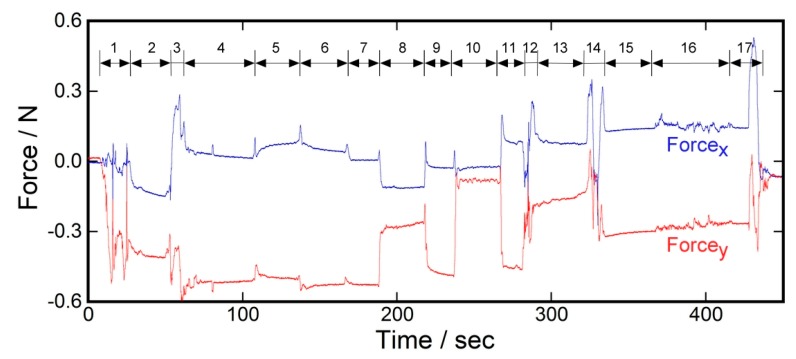
Time course of the measurement result of the demonstrative experiment using the shear force sensor. The test subject changed posture as indicated by the numbers: (1) Sitting down on the wheelchair; (2) Resting; (3) Putting feet on the footrest; (4) Resting; (5) Slouching; (6) Bending backward; (7) Resting; (8) Tilting the body to the left; (9) Resting; (10) Tilting the body to the right; (11) Resting; (12) Slightly detaching the buttocks from the cushion sheet and sliding forward; (13) Bending Backward; (14) Slightly detaching the buttocks from the cushion sheet and sliding to the original position; (15) Resting; (16) Other person pushing the wheelchair randomly (the test subject did nothing); and (17) Standing up.

To demonstrate the usefulness of our sensor for on-site monitoring in the actual environment, a simple experiment using a test subject sitting in a wheelchair was conducted. Figure 11 explains the experimental setup and procedure. A healthy test subject cooperated in this experiment. The basal electrode film of the sensor was attached to the trouser cloth at the ischial tuberosity with double-sided adhesive tape, and was covered with a fragment of cloth using the same adhesive tape to make the sensor surface similar to that of the fabric (Figure 11a). The mobile system with the battery was put into the backside pocket of the wheelchair. Next, the sensor was connected to the mobile system via a cable, and the subject carefully sat on the wheelchair where a cushion seat was placed (Figure 11b). The cable was carefully routed so that the subject did not nip the cable under the buttock. Figure 12 shows an example of the measurement results. The baseline was initialized to be zero just before the measurement. As seen in the chart, the x and y components of the shear force changed according to the various postures of the subject. The increase in the value of Force_x_ meant that the shear force was applied to the buttock toward the forward direction relative to the cushion. Furthermore, the increase in the value of Force_y_ meant that the shear force was applied to the buttock toward the right direction relative to the cushion. The large fluctuations corresponding to No. 1, 12, 14, and 17 were attributed to the attachment and/or detachment of the buttock from the cushion. Putting feet on the footrest (No. 3) also caused a large fluctuation. In general, the movement of the upper body gave a certain displacement to the Force_x_ and Force_y_. In contrast, random passive movements of the wheelchair only made slight changes to them.

**Figure 13:**
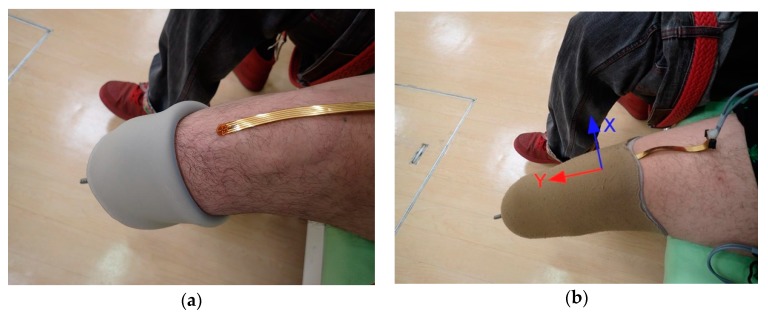
Photographs showing how the sensor was attached to the stump in the prosthesis liner: (**a**) Sensor head of the shear force sensor was attached to the tibial tuberosity with a double-sided adhesive tape, and the measurement started; and (**b**) The prosthesis liner was fully donned.

**Figure 14:**
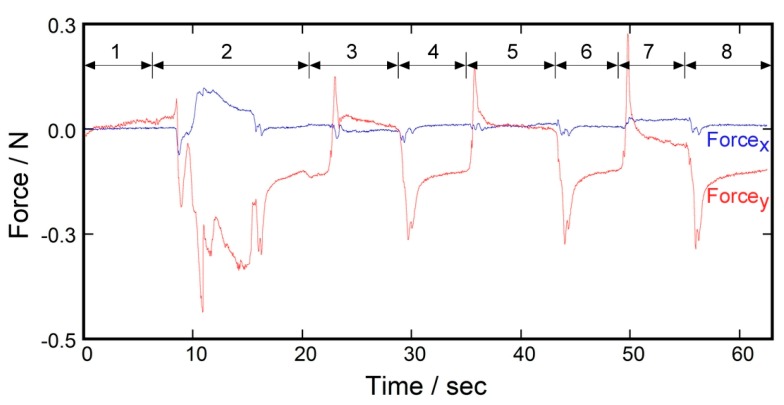
Time course of the measurement result of the prosthetic liner experiment using the shear force sensor. The test subject repeatedly flexed and extended their knee joint during the measurement: (1) Soon after the sensor attachment; (2) Donning the liner to fully cover the knee; (3) Flexion; (4) Extension; (5) Flexion; (6) Extension; (7) Flexion; and (8) Extension.

Another demonstrative experiment was carried out as shown in Figure 13. In this case, shear force between the stump and the prosthetic liner was measured. A person with a transtibial amputation co-operated in this experiment. A shear force sensor was attached on the tibial tuberosity with double-sided adhesive tape before the measurement started. Figure 14 shows the time course of the measurement result. During measurement, the subject flexed and extended one’s knee joint repeatedly. The increase in the value of Force_x_ meant that the shear force was applied to the tibial tuberosity toward the inside of the crotch. The increase in the value of Force_y_ meant that the shear force was applied to the tibial tuberosity toward the downward direction relative to the liner (i.e., the direction in which the liner comes off the stump). As seen in Figure 14, Force_y_ decreased when the knee extended, and returned to the original level when the knee flexed. In contrast, the value of Force_x_ varied little during the experiment. This result suggested that the sensor clearly identified the direction in which the shear force was applied.

## 4. Discussion

The temperature dependence of the raw data meant that the conductivity of the liquid electrolyte was also temperature-dependent. The major factor of electrolyte conductivity is attributed to ion mobility, which is directly related to the liquid viscosity. It is known that liquid viscosity has a negative relationship with temperature, and the relationship is approximated by Andrade’s equation [30]. As a preliminary experiment, we confirmed that the viscosity of the electrolyte used for our sensor also followed this equation. Thus, temperature dependence is inevitable as long as liquid materials are used as a sensor component.

However, this issue with temperature dependence was drastically solved by introducing a simple arithmetic calculation. The potential reason for the solution was that we obtained redundant information from the sensor to produce shear forces-related output. Shear force is a kind of vector value, and is represented by two values corresponding to the orthogonal orientation. Therefore, it is possible to measure the shear force with three electrodes as a minimum constituent. In the case of our sensor, it had five electrodes and two redundant electrodes.

Temperature independence is an important property in practical use, especially in measurements targeting the human body. For example, when inserting a sensor between a human body and an object such as a wheelchair seat or an artificial limb, the temperature influence is exerted on the sensor from both sides. Whether other temperature influences are received can be affected by the position and movement of the body.

Apart from the problem of temperature, the raw sensor output exhibited slight pressure dependency. The reason for this was not exactly clear, because the raw output of all electrodes decreased when pressure was applied. It is plausible that the thickness of the silicone ring decreased and the diameter concurrently increased when pressure was applied. However, in this case, the distance between the central electrode and the L, R, U, D electrodes decreased, causing an increase in conductivity. Therefore, we must consider other possibilities, such as the decrease in the temperature of the sensor by transmitting heat to the weight, or the generation of stray capacitance induced by the metallic weight. However, this was unlikely since our sensor was robust against stray capacitance in principle, and we inserted a thick glass plate between the sensor and the weight.

Moreover, our sensor was resistant to electrostatic induction, as the measurement principle was not potentiometric. Additionally, our sensor was robust against stray capacitance because the major current flowed through the sensor itself via the electrolyte. This enabled the elongation of a flat wiring pattern without a preamplifier or relay amplifier, as shown in Figure 5d. We simply soldered junctions between the adjacent electrode films, as this property was also important for practical use. For example, in the case of a sensor inserted into prosthetics, the sensor head and the wiring part should be sufficiently flat and thin, given that there is only a small gap between the stump and prosthetics.

Therefore, a liquid electrolyte-based sensor was fabricated; however, it seems possible to use any liquid conductor (e.g., ionic liquid and liquid metal instead of liquid electrolyte). The structure of the sensor and the compensation method (the normalization method) for temperature and pressure seems commonly usable for the sensors using any liquid conductors.

It was also necessary to consider the prerequisite of applying this sensor. It should be noted that the data in Figure 8, Figure 9 and Figure 10 were results obtained in an ideal state. As shear force was calculated from sensor responses based on these results, it was assumed that the shear force and pressure were uniformly applied to the sensor surface in the application measurements. When a force was applied to only a part of the sensor surface, it could not necessarily be said that correct measurement results were obtained. The relevance of this prerequisite was determined by the ratio between the size of the sensor and that of the object to be measured. As the diameter of our sensor was 10 mm, we considered the prerequisite to be realistic if we measured the human body. Furthermore, not only our sensors, but most flexible sensors generally have this type of prerequisite.

Moreover, if a force was applied evenly to the sensor surface from a non-vertical direction, it considered that only the shear force component responds. This is because the inner diameter of the rubber ring was made sufficiently smaller than the outer diameter, and the shrinkage of the thickness of the rubber ring was considered to be substantially equal to the force from the oblique, as long as a uniform force was applied.

In this paper, we did not interpret the results of the application experiments, as we have to repeatedly collect such data to confirm reproducibility and to obtain meaningful conclusions for each application field as future work. However, it is emphasized that the experiments suggested that the sensor was a useful tool to obtain on-site monitoring data.

## 5. Conclusions

We introduced the structure of our new type of shear force sensor. Furthermore, we explained the fabrication method of the sensor, the configuration of the measurement system, and the stabilization method of the raw output of the sensor. The sensor output was resistant to the temperature variation and pressure after the stabilization process. Finally, we demonstrated the application using the sensor. Though we have not derived any interpretation from the application measurement, it can be concluded that the sensor is useful for on-site measurement. The sensor was practically useful, as we can use long film-based wiring.
